# Estimating fuzzy measures of deprivation at local level in Tuscany

**DOI:** 10.1007/s11135-023-01679-8

**Published:** 2023-05-27

**Authors:** Federico Crescenzi, Laura Neri

**Affiliations:** 1 Department of Economics, Engineering, Society, Business Organization, Tuscia University, Viterbo, Italy; 2 Department of Economics and Statistics, University of Siena, Siena, Italy

**Keywords:** Deprivation, Multidimensional and fuzzy measures, Small area estimation, Tuscany

## Abstract

In this paper we estimate monetary and non-monetary poverty measures at two sub-regional levels in the region of Tuscany (Italy) using data from the ad-hoc Survey on Vulnerability and Poverty held by Regional Institute from Economic Planning of Tuscany (IRPET). We estimate the percentage of households living in poverty conditions and three supplementary fuzzy measures of poverty regarding deprivation in basic needs and lifestyle, children deprivation, and financial insecurity. The key feature of the survey is that it was carried out after the COVID-19 pandemic, therefore, some of the items collected focus on the subjective perception of poverty eighteen months after the beginning of the pandemic. We assess the quality of these estimates either with initial direct estimates along with their sampling variance, and with a secondary small area estimation when the formers are not sufficiently accurate.

## Introduction

The COVID-19 pandemic outbreak has affected all segments of the population and has been particularly detrimental to members of social groups in the most vulnerable situations. The pandemic has created both a public health crisis and a severe crisis on both the global and national economies and continues to affect populations especially in economic and social areas. Some recent studies have shown that not all the EU felt the pandemic impact on their economies to the same extent: the southern European countries like Spain, Croatia, Greece, and Italy, where the tourism sector plays a relevant role, were the most fragile (EU 2021).

In this paper, we study the economic poverty at regional and sub-regional level after the impact of COVID-19 pandemic in Tuscany, a region in central Italy that heavily founds its economy on exports and various forms of tourism. Tuscany is also a region that, in the face of an underlying cultural homogeneity, has a variety of natural and human environments, as well as structural and economic contrasts across different areas.^1^ Thus, if we only look at regional level, large intraregional disparities are going to be masked.

Understanding poverty at local level is then essential to adequately identify this phenomenon, and to design local policies that aim at mitigating its consequences. Therefore, considering the heterogeneity of the regional territory, we think that it is particularly useful to analyse the phenomenon of economic poverty at sub-regional level. To this purpose, we use data from the Poverty and Vulnerability survey held by Regional Institute for Economic Planning of Tuscany (IRPET) in September 2021.

We consider two hierarchies of sub-regional disaggregation. The first sub-regional level that we consider is a partition of Tuscany into six areas, officially defined by IRPET, obtained as an aggregation of the so-called Local Labour Market Areas. Such grouping of six different areas, refers to the levels of employment, the remuneration of productive factors (labour and capital) and consequently, to different levels of wellbeing. In detail, the six areas are the following: Cities, the urban territories, with an important presence of the tertiary sector to businesses as well as to persons; Made in Italy, manufacturing areas based on the traditional production vocations of textiles, leather, leather goods, furniture, etc.; Other Industry, manufacturing areas not belonging to the Made in Italy sector; Seaside Tourism, coastal territories having a seasonal tourist characterization; Agritourism area, promoting sustainable agriculture and ecological tourism; Internal Areas Northern Apennines, the farthest area from centres, lacking of essential services. The second sub-regional level is the official NUTS-3 level.

When referring to domain measures of poverty and deprivation, we believe that it is important to understand poverty beyond monetary deprivations and to provide reliable evidence to monitor specific domain policy. Therefore, we use the survey data to estimate the percentage of households living in poverty conditions and three fuzzy supplementary measures of poverty based on a fuzzy approach. This approach has a longstanding usage in the literature, see (Betti et al. 2020) for a review in social studies and (Tavares and Betti 2021) for the effect of COVID-19 on poverty in Brazil.

Given the importance of the results for regional policy making, we provide the estimated measures with an estimate of their uncertainty. When the uncertainty around the estimate is too large to be regarded as acceptable, we use small area estimation to obtain more accurate estimates. Empirically, we find out that in some areas/provinces, the estimated coefficient of variation is too large to get a proper level of accuracy of the direct estimates. Nevertheless, we obtain satisfactory results using small area estimation techniques.

The remainder of this paper is structured as follows: Sect. 2 discusses the survey and gives a preliminary regional picture after COVID-19 breakout in Tuscany focusing on preliminary monetary poverty measures and providing a general picture of the perceived economic situation of the inhabitants. Section 3 introduces the fuzzy measures of multi-dimensional poverty. It also introduces small area techniques as a tool to obtain more efficient estimates of poverty measures when the sample size is not enough to guarantee an adequate level of accuracy. Section 4 describes the empirical analysis and the results obtained at area and NUTS-3 level. It shows both direct and small area estimates. Section 5 concludes the paper and provides directions for further research work.

## The survey and the regional scenario

The sample survey “Indagine sulla Vulnerabilità alla Povertà” was conducted in September 2021 by the Regional Institute for Economic Planning of Tuscany. Its focus is on the economic and social features of the Tuscan households, with particular attention to the current economic situation and prospects. A sample size of 2512 households has been drawn to achieve representativity at NUTS-3 level. Interviews were conducted by C.A.T.I.^2^ and C.A.M.I.^3^ methods, interviewing one adult household member. After a weighting procedure, the sample totals conform to the population totals as regards gender and age groups. As regards item nonresponses, missing data have been imputed by deductive imputations based on logical or mathematical relationships between the variables, where it was possible. Thirteen units having missing values for all the eleven deprivation indicators collected for the present situation and for the pre-COVID were discarded. Therefore, the valid units for the analysis are 2499. Item nonresponses relative to some quantitative and qualitative variables were imputed with stochastic imputation methods, assuming fully conditional specification.^4^ The largest number of missing values (14,5%) was registered for the only question adopted to collect the approximative monthly total net household income. The approximative values collected may lead to a bracket distribution, as follows: [0–600 euro]; [600–700]; [700–900]; [900–1100]; [1100–1300]; [1300–1500]; [1500–1700]; [1700–1900]; [1900–2250]; [2250–2750]; [2750–3500]; [3500–4500]; [4500–5500]; [5500–6500]; [6500–8000]; [8000–10,000]; [10,000 or more]. Continuous values within each bracket have been imputed considering the kernel density estimate of the empirical distribution of the variable. Based on the total household disposable income, we retrieved the equivalized income using the OECD-modified equivalence scale. The poverty line was taken as the 60% of the median of such equivalised income distribution among the 5523 individuals present in the valid 2499 interviewed households.

According to IRPET^5^ the households in absolute poverty in Tuscany went from 3.2% to 3.3% (a negligible increasing) thanks to the interventions put in place to protect families to contain the effects of the pandemic. Referring to a relative measurement approach and using the Eurostat-type poverty line, we estimated the head count ratio at regional level to be equal to 11.58%.^6^

In order to have a general picture of the perceived economic situation of the inhabitants, Table 1 shows some descriptive results at regional level. In particular, the data collected from the question: “Taking into account your actual income, how can your household make ends meet? With great difficulty/some difficulty/difficulty/fairly easily/easily/very easily?” (Ravallion 2014, 2015) show that more than half of the households (53,06%) make ends meet facing at least with “some difficulty”.

**Table 1:** Subjective poverty (2021): ability to make ends meet (%)

Making ends meet…	%
With great difficulty	7.15
Difficulty	11.40
With some difficulty	34.51
Fairly easily	30.07
Easily	14.61
Very easily	2.26

Moreover, Table 2 shows that for about 33% of the households the economic situation at least “slightly worsened” with respect to the pre-pandemic period (2019).

**Table 2:** Comparing current economic situation (2021) with respect to 2019

The economic situation has…	%
Improved	5.66
Unchanged	61.46
Slightly worsened	23.5
Greatly worsened	9.38

**Fig. 1:**
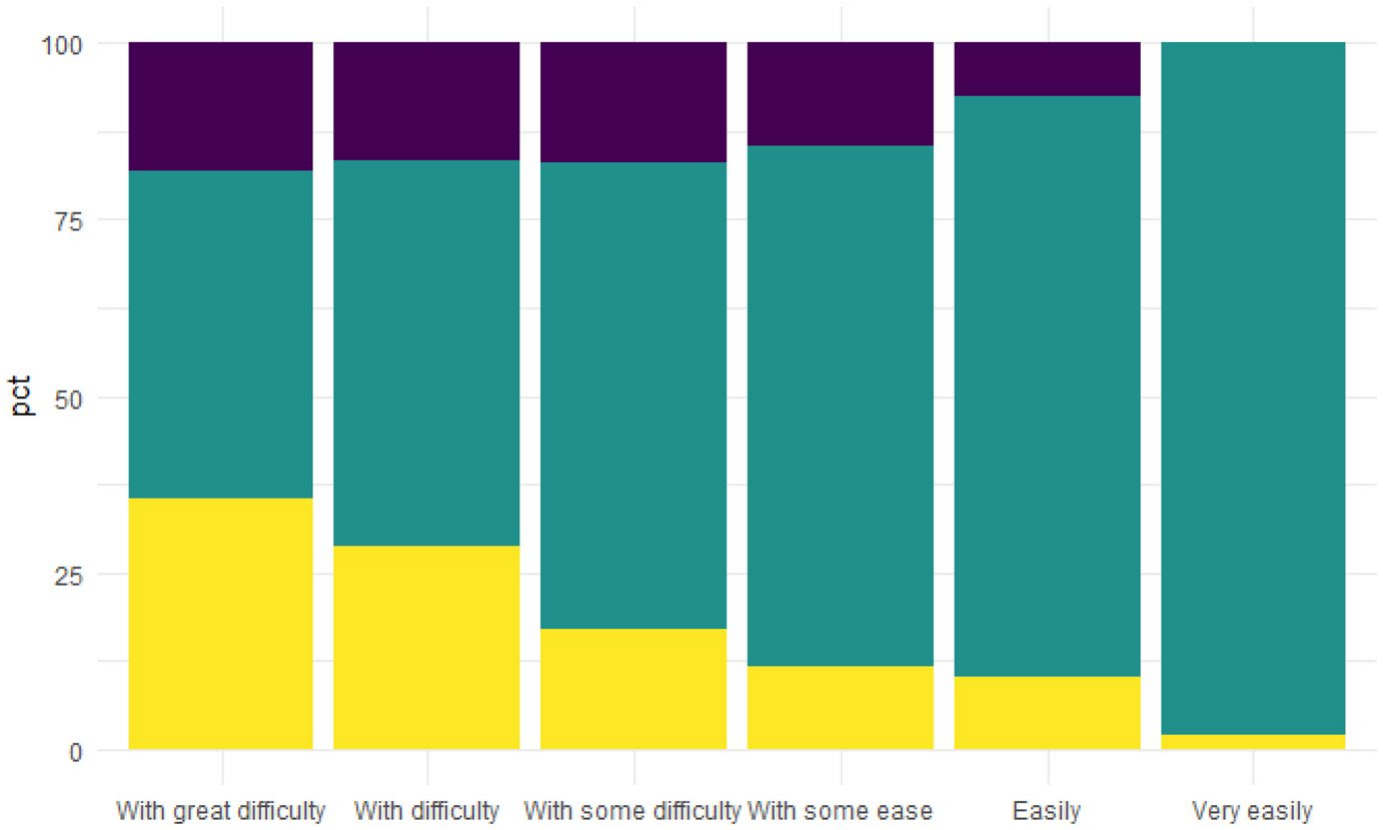
Economic expectations conditional on the current ability to make ends meet (2021). Improving expectations (dark blue), unchanged expectations (green), worsening expectations (yellow). (Color figure online)

As of what the households expect for the coming twelve months, by analysing the distribution of the households expectations, conditioned to the “ability to make ends meet”, we notice that the difficulties to make ends meet increase as it does the percentage of households expecting worsening for the next months (see Fig. 1). Thus, the actual difficulties, even if strongly influenced by the contingent situation of the pandemic are perceived as a middle/long term situation.

Although monetary poverty can capture a household’s ability to meet critical situation like the pandemic one, surely it does not capture all forms of deprivation. For this reason, our analysis considers also non-monetary multidimensional measures of poverty.

## Methods

### Fuzzy measures of multidimensional poverty

The fuzzy sets approach is a valid instrument to measure multidimensional poverty and it offers the additional advantage of overcoming the use of unavoidably arbitrary poverty thresholds. In such a way, it avoids extreme simplifications and loss of statistical information, deriving from the rigid poor/non poor dichotomy. The fuzziness is accounted for via a poverty membership function, measured on a scale from 0 to 1—whereby 1 means full membership to the set of the poor and 0 full non-membership—which allows to deal with such a blurry (as opposed to sharp) vision of the poverty concept. The conventional classification into a rigid dichotomy according to the traditional approach to poverty can then be viewed as a special case of the fuzzy conceptualization of poverty, where the membership function equals 1 for those below the poverty line and 0 for those above the poverty line.

There are several advantages of treating poverty and deprivation as a matter of degree, applicable to all members of the population, rather than as simply a ‘yes–no’ state. First, Non-monetary deprivation depends on forced non-access to various facilities or possessions determining the basic conditions of life. An individual may have access to some but not to others. Hence, non-monetary deprivation is inherently a matter of degree, and some quantitative approach such as the present one is essential. Second, further insights into the relative income situations of individuals and groups can be obtained by incorporating into the poverty rates a measure of the actual levels of incomes received, particularly at the lower end of the income distribution. Third, the fuzzy approach provides more robust indicators of poverty (or more generally, of deprivation in multiple dimensions) in the *longitudinal* context. The conventional approach measures mobility, simply in terms of movements across some designated poverty line, does not reflect the actual magnitude of the changes affecting individuals at all points in the distribution. Consequently, the degree of mobility of persons near the chosen line tends to be over-emphasised, while that of persons far from that line largely ignored.

Betti and Verma (2008) proposed the following fuzzy measures based on the seminal contributions of (Cerioli and Zani 1990; Cheli and Lemmi 1995); then furtherly elaborated in (Betti et al. 2015). In the generalised form, the membership function of monetary or non-monetary deprivation is defined for any individual of rank $j$ in the ascending income distribution as:

1 $${\mu }_{j,K}={\left(\frac{\sum_{\gamma =j+1 }^{n}{w}_{y}|{s}_{\gamma }>{s}_{j}}{\sum_{ \gamma =2}^{n}{w}_{\gamma }|{s}_{\gamma }>{s}_{1}}\right)}^{\alpha -1}\left(\frac{\sum_{\gamma =j+1 }^{n}{{s}_{\gamma }w}_{y}|{s}_{\gamma }>{s}_{j}}{\sum_{ \gamma =2}^{n}{{s}_{\gamma }w}_{\gamma }|{s}_{\gamma }>{s}_{1}}\right)$$

where $s$ is the overall score in the non-monetary deprivation (defined below), ${\mathrm{w}}_{\upgamma }$ is the sample weight of individual of rank $\upgamma$ and $\alpha$ is a parameter to be estimated on the data (see step 6 below). It is possible to reformulate formula (1) as:

2 $${\mu }_{j,K}={\left(1-{F}_{j,K}\right)}^{{\alpha }_{k}-1}(1-{L}_{j,K})$$

where $\left(1-{\mathrm{F}}_{\mathrm{j},\mathrm{K}}\right)$ is the proportion of individuals less poor (less deprived) than the person concerned and ${\mathrm{L}}_{\mathrm{j},\mathrm{K}}$ represents the value of the Lorenz curve for individual j.

As regards the computation of the fuzzy supplementary (FS) measures, the general procedure to quantify and put together diverse indicators of deprivation is based on the following steps:

1. Identification of items of deprivation to be included in the analysis.
2. Transformation of the items in the $[\mathrm{0,1}]$ interval.
3. Exploratory and confirmatory factor analysis to identify measures of deprivation.
4. Calculation of weights of individual items of deprivation within each dimension.
5. Calculation of scores within each dimension.

Calculation of an overall score and parameter $\alpha$.

Construction of the fuzzy deprivation measures separately in each dimension, taking their simple average as a measure of overall non-monetary (supplementary) deprivation.

We remind to Betti et al. (2015) for more details on the steps 2 and 4 above. Broadly speaking, Step 2 maps categorical items to the [0,1] interval using the distribution function of the item. We do not need this step because the items that we consider are already in [0,1]. Step 4 calculates a weight for each item based on the coefficient of variation of the transformed item (Step 2) and the correlation of the item with those in the same dimension found in Step 3. Let ${s}_{j,h}$ be the score of the unit $i$ computed for for each dimension $h$ in step 5. The overall score ${s}_{j}$ is calculated as a simple average over the $m$ dimensions

3 $${s}_{j}=\sum_{h=1}^{m}\frac{{s}_{j,h}}{m}$$

As in step 2, to transform a generic item into the $[\mathrm{0,1}]$ interval we remind the reader to (Betti et al. (2015). In the case of dichotomous items, like those used in this paper, the deprivations score $s$ is 0 for non-deprivation and 1 otherwise.

As of step 6 and the calculation of the parameter $\alpha$ this is done by solving the non-linear equation:

4 $${\mathbb{E}}\left[{\mu }_{k}\right]=\widehat{HCR}$$

where $${\mathbb{E}}\left[{\mu }_{k}\right]$$ denotes the expectation of the fuzzy membership function (in dimension $$k)$$ with respect to the probability measure induced by the sampling design and  $$\widehat{HCR}$$ is the estimated Head Count Ratio using survey data.

### Small area estimation

Sample surveys are widely used in practice to provide estimates not only for the whole target population of interest, but also for a variety of its subsets or subdomains. These can be either geographical like areas, counties, districts, or any sub-population, such that the survey is usually designed to be representative at a higher hierarchy level. In general, it is common to refer to estimates that use only sample weights as *direct* estimates, that is, a direct estimator is design-based. Sometimes though, these direct estimates may suffer from high variance if the number of sample units in the subdomain is not large enough. The term *small area* is used in the literature to refer to these subdomains. The idea behind Small Area Estimation (SAE) is to borrow strength from auxiliary variables to obtain indirect estimators that may exhibit a lower mean squared error than that of the direct estimator. Many small area models have been proposed in the literature (Rao and Molina 2015), in this paper we make use of the Fay-Herriot model (FH) (Fay and Herriot 1979). The setting is as follows: let ${\widehat{\theta }}_{i}$ be an unbiased direct estimator of the $i$-th area parameter ${\theta }_{i}$ so that

5 $${\widehat{\theta }}_{i}={\theta }_{i}+{e}_{i}$$

where ${e}_{i}{\sim }_{i.i.d.} N(0, {\psi }_{i}).$ Then, let

6 $${\theta }_{i}={z}_{i}^{^{\prime}}\beta +{v}_{i}, i=1,\dots , M$$

where M is the number of small areas, ${z}_{i}$ is a vector of area level covariates and ${v}_{i}\sim N(0, {\sigma }_{v}^{2})$, so that by combining the two equations above we obtain

7 $${\widehat{\theta }}_{i}^{F}={z}_{i}^{^{\prime}}\beta +{v}_{i}+{e}_{i}, i=\mathrm{1,2},\dots ., M.$$

The Best Linear Unbiased Predictor (BLUP) estimator of ${\theta }_{i}$ is then given by

8 $${\widetilde{\theta }}_{i}^{F}={\gamma }_{i} {\widehat{\theta }}_{i}+\left(1-{\gamma }_{i}\right){z}_{i}^{^{\prime}}\widetilde{\beta }$$

where ${\gamma }_{i}={\sigma }_{v}^{2}/({\psi }_{i}+{\sigma }_{v}^{2}$) and  $\widetilde{\beta }$ is the Best Linear Unbiased Estimator (BLUE) of $\beta$. As the BLUP estimator depends on the unknown parameter ${\sigma }_{v}^{2}$, the empirical BLUP (EBLUP) is obtained by replacing it with a consistent estimator ${\widehat{\sigma }}_{v}^{2}$, so that the EBLUP estimator of  ${\widehat{\theta }}_{i}$ turns out to be

9 $${\widehat{\theta }}_{i}^{F}={\widehat{\gamma }}_{i} {\widehat{\theta }}_{i}+\left(1-{\widehat{\gamma }}_{i}\right){z}_{i}^{^{\prime}}\widehat{\beta }$$

where $\widehat{\beta }$ is the BLUP estimator of $\beta$ having plugged in the estimator of ${\sigma }_{v}^{2}$. Thus, the small area estimator under the FH model is a linear combination of the direct estimator  ${\widehat{\theta }}_{i}$ and the synthetic estimator from the model in Eq. (8), where greater the variance of the direct estimator greater the weight attached to the synthetic estimate. Concerning the details about the estimation of the mean squared error of the FH estimator, for the sake of brevity of exposition, we limit to say that there exists substantially to approaches, namely: direct computation or the bootstrap. For more details on the two we remind the reader to (Rao and Molina 2015).

## Measurement issue and empirical findings

### Measurement issue: fuzzy measures computation

To compute the fuzzy supplementary measure of poverty, we consider eleven binary deprivation indicators focusing on households’ current situation (September 2021).^7^ The indicators are based on standard questions regarding: affordability to eat nutritional meals, to keep household adequately warm, to cover costs for health, to cover costs for 1 week holiday, to cover costs for cinema, theatre, eating out once a month, to cover costs for transport, for children clothes, toys, specific children food); to cover costs for education such as taxes, books and materials and finally and then ability to cope with unexpected expenses of different amount.

**Fig. 2:**
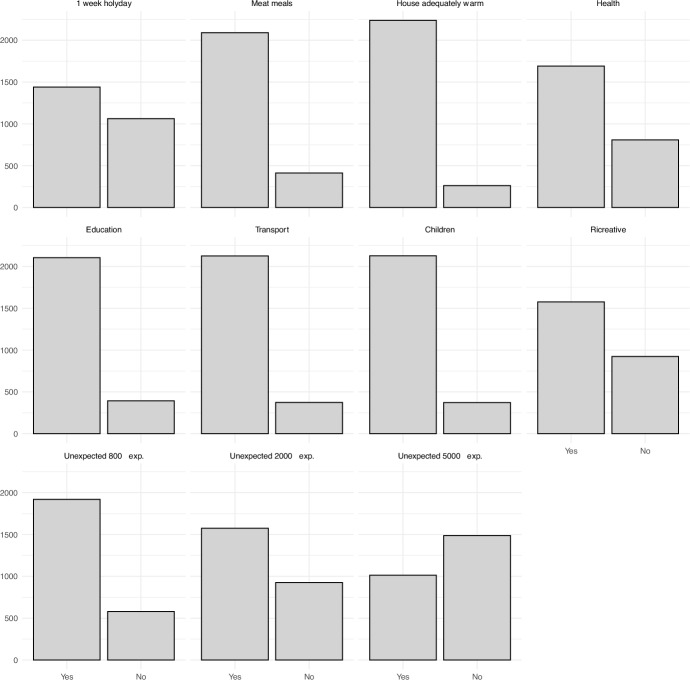
Item frequency distributions “Can you afford…?”

The frequency distribution of these items is shown in Fig. 2. Among these, we notice that more than 1000 households (about 42%) cannot afford a one-week holiday. Also, most households cannot afford an unexpected 5000€ expense. Table 3 shows the matrix of tetrachoric correlations between items. Although, the correlations are moderate in general, the group of items that regard the possibility to cope with unexpected expenses are highly correlated. Also, it is reasonable that households that tend to spend more in Children care are also those that have higher expenditure in education. Interestingly, there is also a substantial correlation between the possibility of affording one-week holyday and expenditure in recreative activities. As of step 2 of constructing FS measures, there is no need to rescale the items, as they are already in [0,1].

**Table 3:** Tetrachoric correlation matrix between items

	Afford one week holiday	Meals with meat	Household adequately warm	Health	Education	Transport	Children	Recreative	Unexpected €800 expense	Unexpected €2000	Unexpected €5000 expense
Afford one week holiday	**1.00**	0.65	0.52	0.59	0.61	0.52	0.62	**0.77**	0.63	0.63	0.61
Meals with meat	0.65	**1.00**	0.53	0.51	0.57	0.48	0.58	0.66	0.53	0.53	0.56
Household adequately warm	0.52	0.53	**1.00**	0.43	0.48	0.41	0.51	0.46	0.36	0.34	0.37
Health	0.59	0.51	0.43	**1.00**	0.56	0.58	0.55	0.54	0.45	0.49	0.51
Education	0.61	0.57	0.48	0.56	**1.00**	0.70	**0.99**	0.61	0.50	0.51	0.50
Transport	0.52	0.48	0.41	0.58	0.70	**1.00**	0.66	0.54	0.45	0.46	0.45
Children	0.62	0.58	0.51	0.55	**0.99**	0.66	**1.00**	0.63	0.51	0.51	0.51
Recreative	**0.77**	0.66	0.46	0.54	0.61	0.54	0.63	**1.00**	0.53	0.54	0.57
Unexpected €800 expense	0.63	0.53	0.36	0.45	0.50	0.45	0.51	0.53	**1.00**	**0.99**	**0.93**
Unexpected €2000 expense	0.63	0.53	0.34	0.49	0.51	0.46	0.51	0.54	**0.99**	**1.00**	**0.97**
Unexpected €5000 expense	0.61	0.56	0.37	0.51	0.50	0.45	0.51	0.57	**0.93**	**0.97**	**1.00**

The dimensions of deprivation have been further investigated by an exploratory factor analysis (see Table 3) to identify the hidden dimensions of the multidimensional poverty (step 3). The three factors extracted account for 53% of the total variance.

The Kaiser–Meyer–Olkin measure of sampling adequacy (0.81) indicates that the factor analysis method is suitable for the collected data (Table 4).

**Table 4:** Factor loadings and variance explained of the Fuzzy Supplementary (FS) measures

Variable	FS3	FS2	FS1	h2*	u2**	Com***
Meals with meat	0.67	0.14	0.29	0.55	0.453	1.5
Afford one week holiday	0.47	0.21	0.19	0.30	0.701	1.7
Household adequately warm	0.33	0.17	0.09	0.14	0.855	1.7
Health	0.45	0.20	0.21	0.29	0.713	1.8
Education	0.25	0.94	0.12	0.95	0.045	1.2
Transport	0.33	0.36	0.15	0.26	0.742	2.4
Children	0.27	0.86	0.13	0.84	0.161	1.2
Ricreative	0.67	0.17	0.21	0.52	0.476	1.3
Unexpected €800 expense	0.26	0.15	0.67	0.53	0.466	1.4
Unexpected €2000 expense	0.20	0.12	0.97	1.00	0.005	1.1
Unexpected €5000 expense	0.33	0.10	0.57	0.45	0.554	1.7
SS loadings	1.983	1.956	1.888			
Proportion Var	0.180	0.178	0.172			
Cumulative Var	0.180	0.358	0.530			

RMSEA = 0.044. Kaiser–Meyer–Olkin overall MSA = 0.81

^*^Amount of variance in the item/variable explained by the (retained) factors

^**^Residual (1-h2)

^***^Item Complexity

Estimates are obtained using maximum likelihood estimation and varimax rotation. The data refers to year 2021

Successively the latent structure identified has been validated using a confirmatory factor analysis (CFA). The interpretation of these results is shown in Table 5. The dimension (FS3) refers to “Inadequate Basic needs and non-inclusive lifestyle”. Indeed, the indicators that mostly contribute to this dimension, all refer to the lack of possibility of satisfying basic needs and the possibility to live with an inclusive lifestyle.

**Table 5:** Dimensions and indicators of the Fuzzy Supplementary (FS) measures (2021)

Dimensions	Indicators
FS3 Inadequate basic needs and non-inclusive lifestyle	Meals with meat or fish // Household adequately warm // cover costs for health // cover costs for 1 week holiday// cover costs for cinema, theatre, eating out once a month
FS2 Children specific deprivation	Costs for: transport// children (clothes, toys, child's food)// education (taxes, books and materials)
FS1 Financial insecurity	Inability to cope with unexpected expenses: 5000, 2000, 800 Euros

The second dimension shows high factor loadings for items regarding expenditure for children needs and education. Therefore, we interpret it as an indicator of “Children specific deprivation” (see Carraro and Ferrone 2020; Benedetti et al. 2020 for related studies on this topic).

The dimension FS1 involves expenditure inability to cope with unexpected expenses, therefore we interpret it as an indicator of “Financial insecurity”. Indeed, we use to say that households are financially insecure if they have not enough assets to face an event that decreases incomes or increases expenses (Prieto 2022).

Overall, the item complexity of each item is close to 1 (below two for 10 out 11 items) suggesting that the items reflect approximately one construct each. The only item that has an item complexity value above two is that of covering costs of transports. The reason of this may be in that this item is approximately correlated to all other items in the same extent, so that it is less clear to what dimension it belongs more.

Then, we calculate weights (step 4) and scores (step 5) withing each dimension and solve the non-linear equation in Formula 4 using the expectation with respect to the probability measure induced by the sampling design (step 6). This step is done using the average in Formula 3. Having obtained the value of $\alpha$, we use it to calculate Formula 2 in each dimension separately.

### Measurement issue: small area level estimation

As said in the introduction of the paper. we consider the IRPET’s territory subdivision of Tuscany into the six areas (Table 6) and NUTS-3 (Table 7). These tables suggest that in some domains the sample size is likely too small to produce reliable estimates at local level. In fact, the reliability of the estimated parameter is often related to the sampling variability.

**Table 6:** Sample sizes by geographical areas (2021)

Area	Sample size
5. Agritourists	67
6. Internal Areas North-Apennine	72
4. Seaside Tourism	270
3. Other Industry	614
2. Made in Italy	725
1. Cities	751

**Table 7:** Sample size by Province (2021)

NUTS-3	Sample size
Prato	83
Massa	94
Livorno	164
Grosseto	166
Pistoia	175
Arezzo	207
Lucca	263
Siena	320
Pisa	336
Firenze	691

The results from direct estimation shown in the section below confirm this intuition and, consequently, the need to use SAE methods. To apply such methods, we first estimate sampling errors for each domain using the bootstrap. Next, we review possible (local) data sources suitable to find auxiliary variables at small area level. The auxiliary variables that we use (see Formula 7) come from administrative data source (IRPET), therefore they are measured without error (see Arima et al. 2015; Bell et al. 2019 for a dissertation on when covariates are measured with errors).

To assess the accuracy of the results based on the survey data we use the coefficient of variation as it is a standardized measure of the sampling variability.^8^ Statistics Canada,^9^ provides guidelines for publication related to the uncertainty of estimates specifying the following levels of data quality: excellent (0–5%); very good (5–15%); good (15–25%); acceptable (25–35%); (> 35%) use with caution. Nevertheless, many Official Statistical Agencies do not publish estimates with CV higher than 20%.

### Empirical findings

#### Area level estimation

This section reports the estimates of the monetary and non-monetary poverty measures at area and NUTS-3 level using the survey data. The estimation of the percentage of households living in poverty conditions has used the imputed income described in Sect. 2. All SAE estimates are compared with the corresponding direct estimates either in terms of their point estimates and coefficients of variations (CVs). Small area etimates were obtained using the sae R-package (Molina and Marhuenda, 2015).

**Fig. 3:**
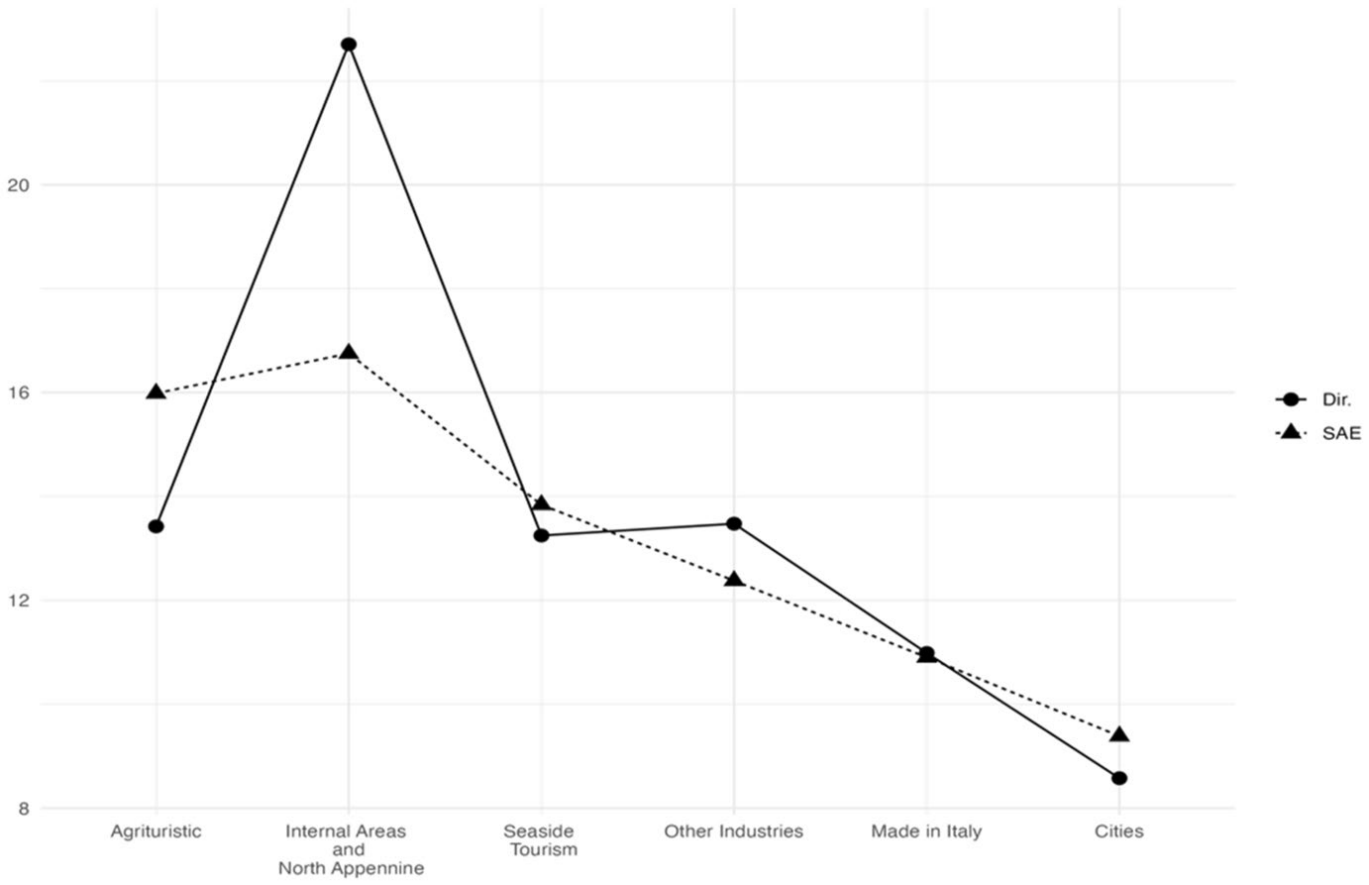
EBLUPs based on FH model of HCR by areas (2021). Areas are sorted by increasing sample size

**Fig. 4:**
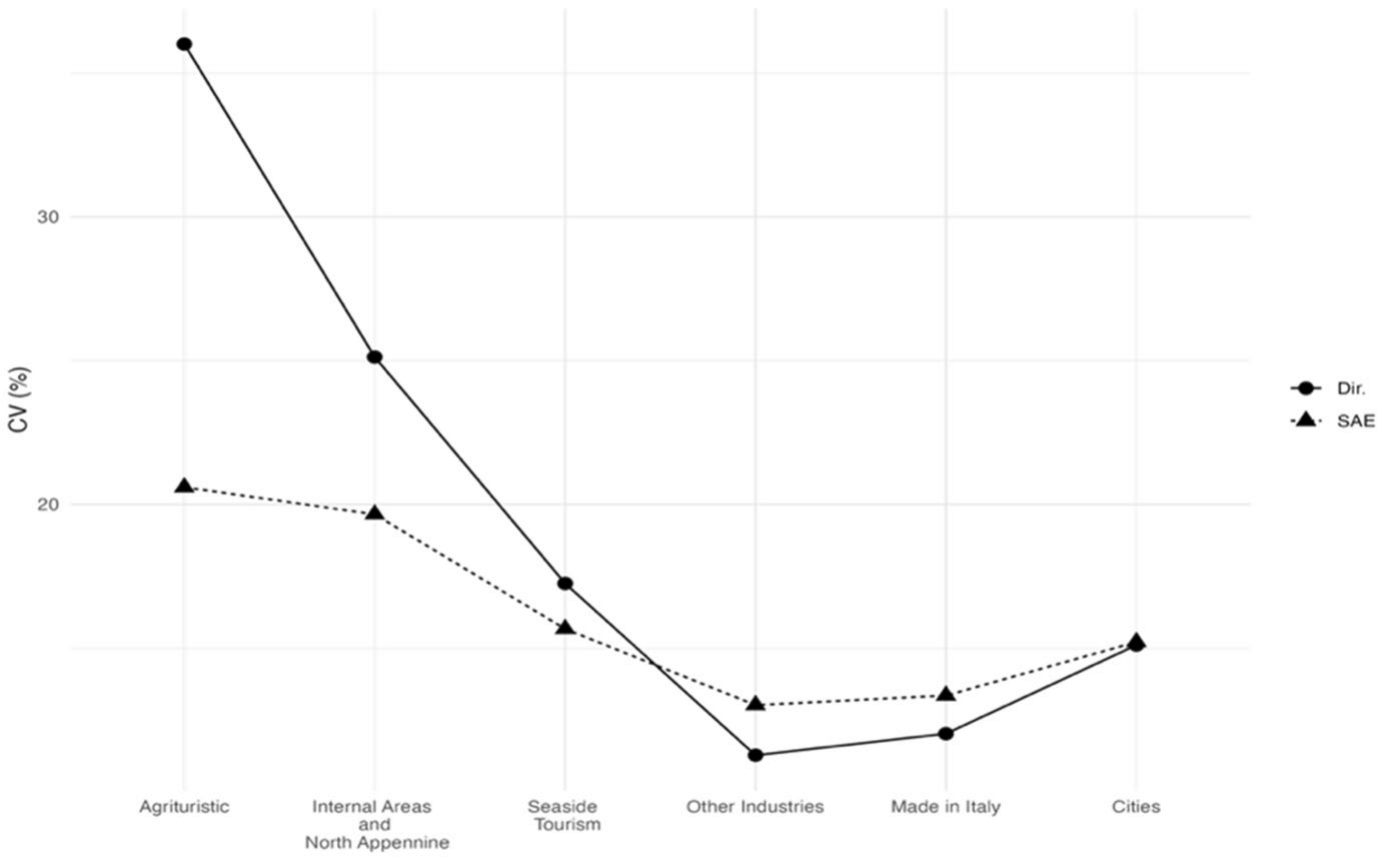
CVs of EBLUPs and direct estimators for each area (2021). Areas are sorted by increasing sample size

Starting with the six areas, the estimated HCRs (Fig. 3) show that “Cities” and “Made in Italy” have less households living in poverty conditions while “Agritourists” and “Internal Areas” are the poorest areas. Interestingly, the SAE estimate of “Internal Areas” and “North-Apennines” revises downwards the direct estimates. Figure 4 shows that the gains in efficiency of the EBLUPs tend to be larger for areas with smaller sample sizes. Thus, EBLUPs based on FH model seem more reliable than direct estimators. To obtain these estimates we used the following auxiliary variables: the percentage of people employed (for HCR). and the percentage of people receiving citizenship retirement benefits (for FS1. FS2. FS3).

**Fig. 5:**
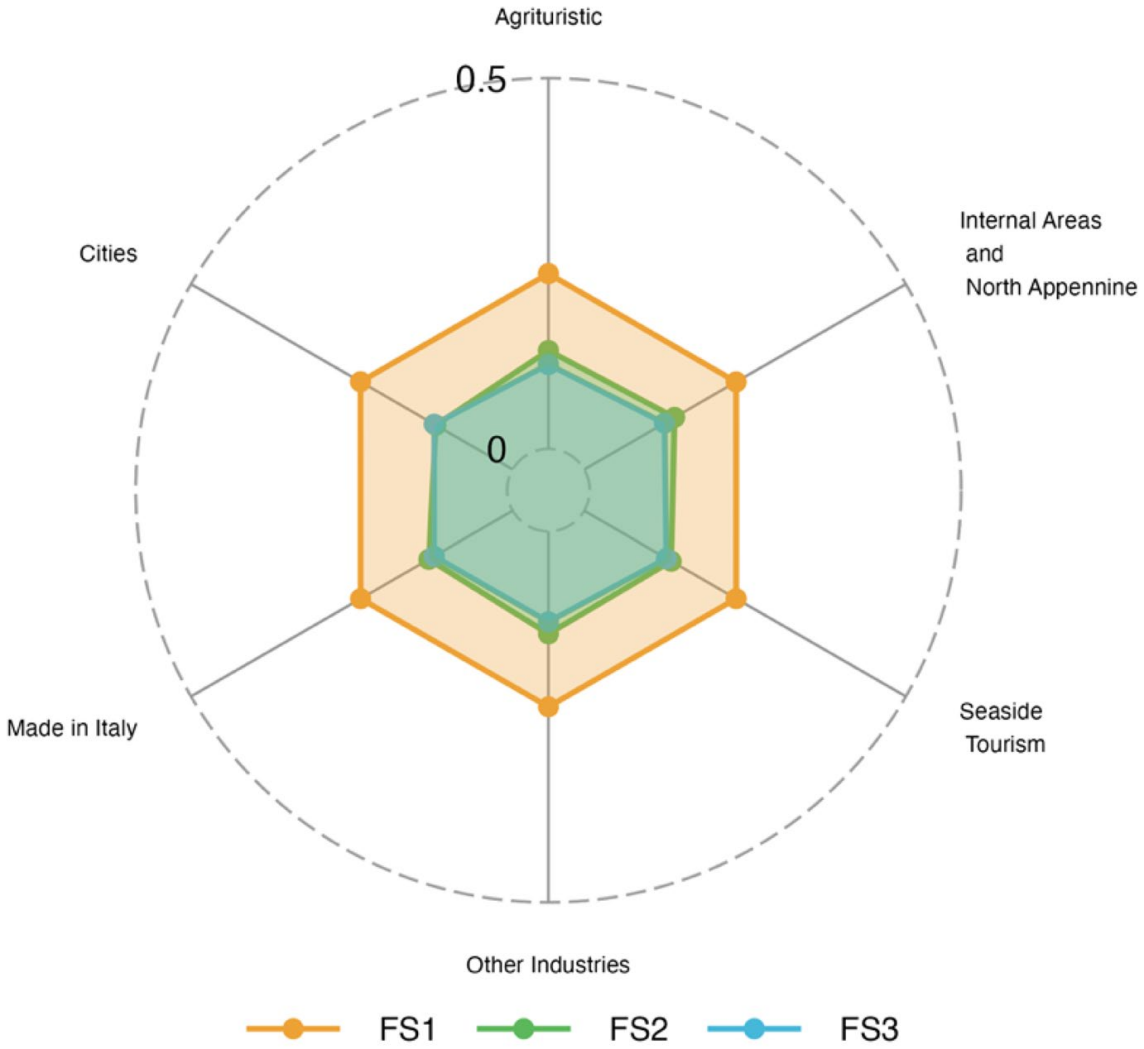
Fuzzy Supplementary (FS) poverty measures (2021). Area Level

Financial insecurity (FS1) is the most dominant dimension of poverty in all areas considered (Fig. 5), and it is followed in a less extent by children-specific vulnerability. Interestingly, the “Cities” area is the one that experiences much less the dimension of financial insecurity, maybe, this is due to that households have enough assets to face an event, like the pandemic, that in many cases decreases incomes. This result is coherent with the HCR (Table 8) as for “Cities” this is much lower than in other areas.

**Table 8:** Area Level final estimates (2021)

Stratum	Head count ratio	FS1	FS2	FS3
Agrituristic	15.982*	0.236*	0.130*	0.122*
	(3.291)	(0.031)	(0.022)	(0.024)
Cities	8.578	0.204	0.117	0.099
	(1.297)	(0.031)	(0.022)	(0.017)
Internal Areas	16.750*	0.237*	0.120*	0.122*
	(3.292)	(0.075)	(0.053)	(0.048)
Made in Italy	10.986	0.255	0.148	0.129
	(1.322)	(0.034)	(0.025)	(0.019)
Seaside Tourism	13.387*	0.236*	0.138*	0.122*
	(2.170)	(0.032)	(0.025)	(0.022)
Other Industry	13.472	0.236	0.149	0.146
	(1.521)	(0.037)	(0.027)	(0.022)

The * denotes model-based estimates. Root mean squared error in parenthesis

**Fig. 6:**
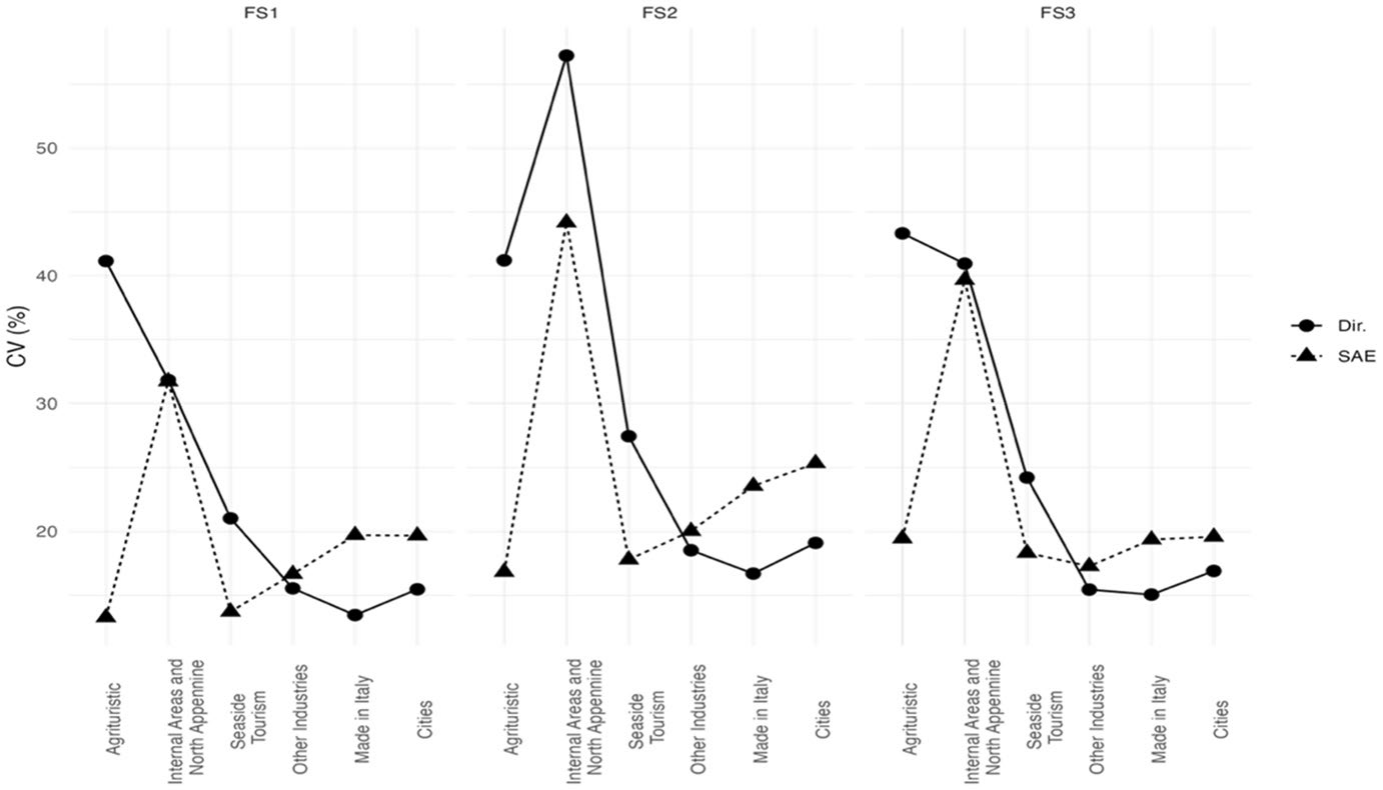
CVs of EBLUPs and direct estimators for each area (2021). Areas are sorted by increasing sample size

Figure 6 show that we obtain the major gains in efficiencies in the areas having a lower sample size although in the most sampled areas. The mean squared error of the small area estimate is sometimes larger than that of the direct estimate. However, this is not necessarily a problem as the estimates in these areas have good quality.

Table 8 reports the final estimates for the areas considered along with their root mean squared errors. For each area, we report the most efficient estimate between the direct estimate and the model-based estimate.

#### NUTS-3 level estimation

As regards NUTS-3 level results, we notice (see Table 7) that Prato and Massa-Carrara are the provinces with the smallest sample sizes, while Florence and Pisa have the two greatest ones.

**Fig. 7:**
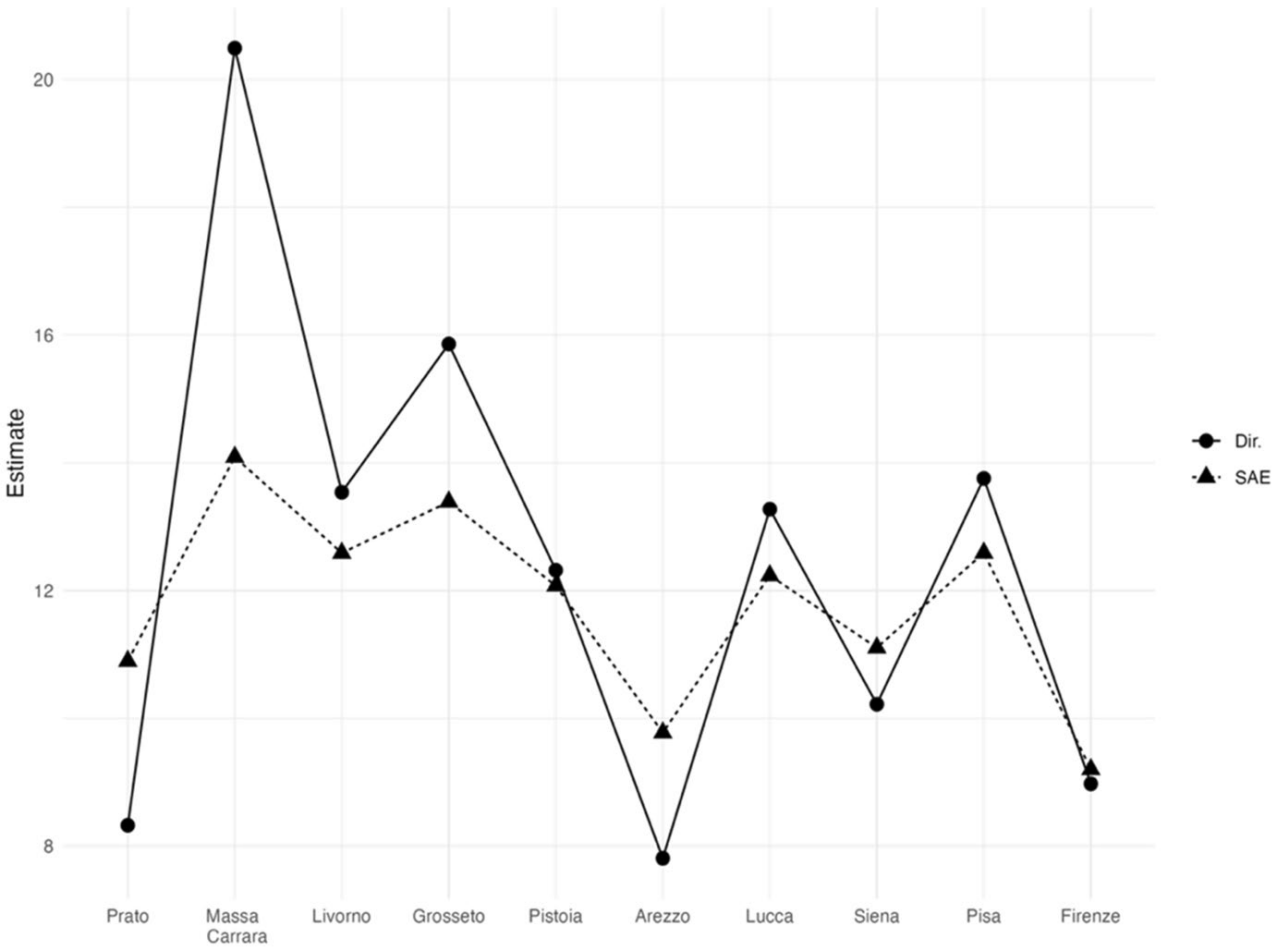
EBLUPs based on FH model and direct area estimates of HCR by provinces (2021). Areas are sorted by increasing sample size. (Color figure online)

**Fig. 8:**
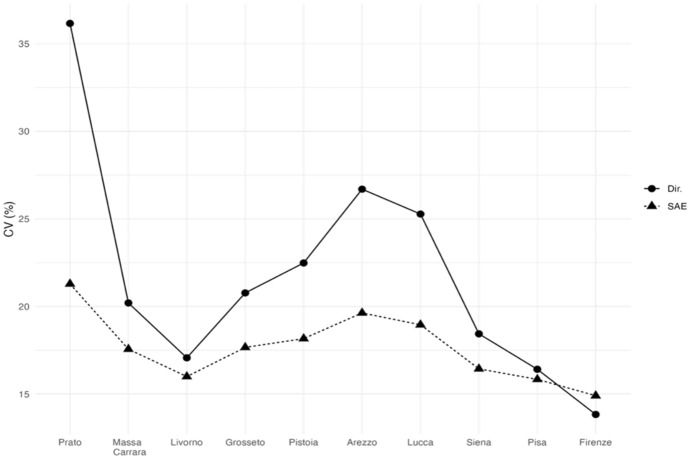
CVs of EBLUPs and direct estimators for each area (2021). Areas are sorted by increasing sample size

In Fig. 7, the estimated HCR is tracked, and we can observe that EBLUPs track direct estimators but are much less volatile. According to the EBLUP HCRs we observe that Siena, Firenze, Prato and Arezzo show figures below the regional HCR and that Massa and Grosseto present much greater figures. At this level of territorial disaggregation. Figure 8 shows that only three provinces (Prato, Arezzo, and Siena) have HCR direct estimates with CVs over 25% (acceptable), whereas the CVs of the SAE estimates do not exceed 22% for any of the areas (very good or good quality).

**Fig. 9:**
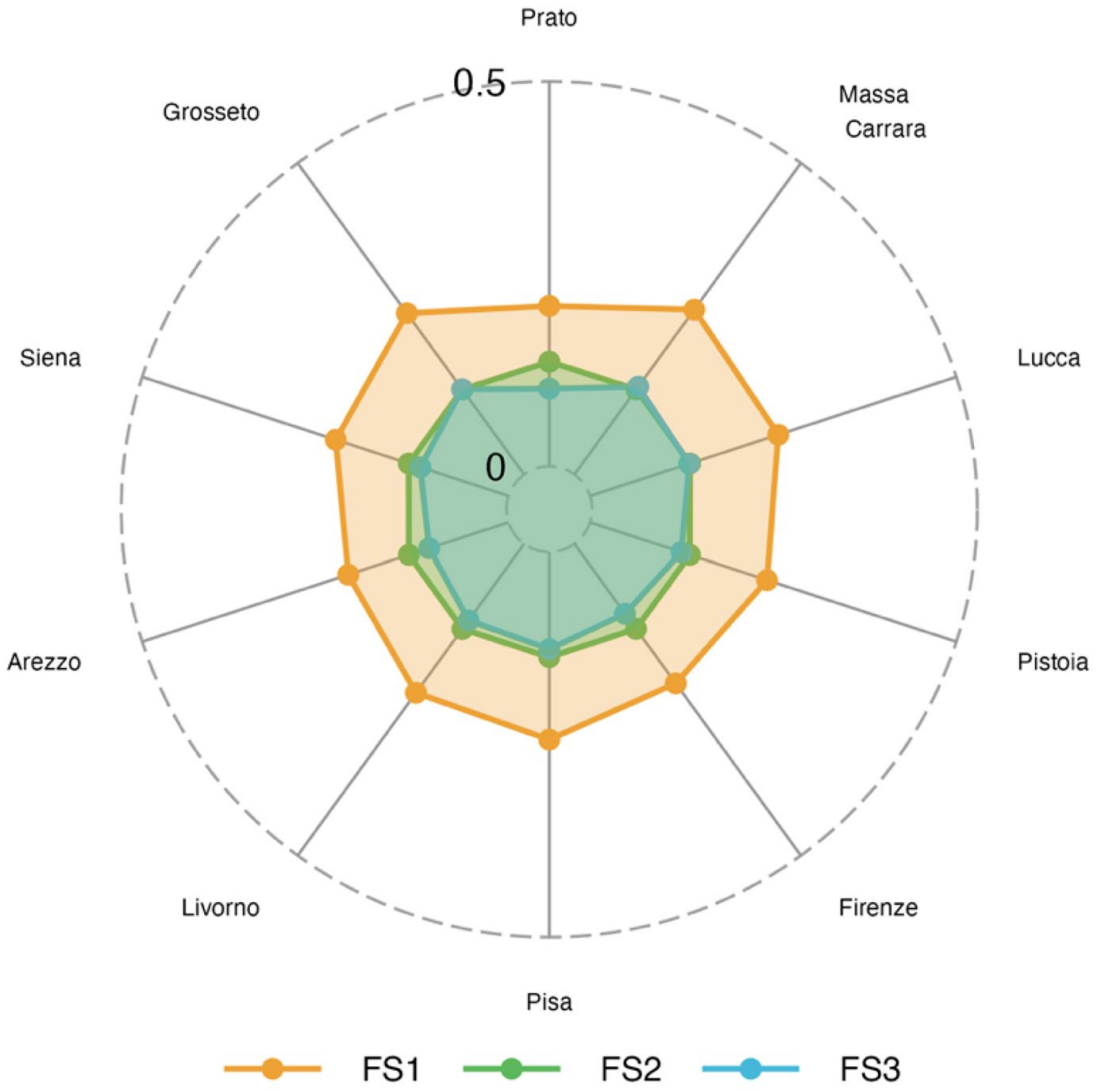
Fuzzy Supplementary (FS) poverty measures (2021). Province Level

Regarding non-monetary poverty (see Fig. 9), financial insecurity (FS1) is the dimension that dominates in all the provinces considered. In this dimension, the less deprived province is Prato, followed by Arezzo. The ten provinces show similar deprivation measures as regards FS2. FS3 is a dimension that shows the lowest values and interestingly, it is particularly contained in the provinces of Arezzo and Prato. These provinces together with Florence are at the same time those that have the lowest values of the monetary poverty (Table 9). Moreover, in Florence, where the tourism sector plays a key role, the pandemic impacted more than in Arezzo and Prato with economies largely based on manufacturing sector.

**Table 9:** NUTS-3 final estimates (2021)

NUTS-3	Head count ratio	FS1	FS2	FS3
Prato	10.896*	0.208*	0.136*	0.101*
	(2.320)	(0.041)	(0.029)	(0.023)
Massa	14.090*	0.265*	0.137*	0.141*
	(2.474)	(0.044)	(0.031)	(0.024)
Lucca	12.588*	0.257*	0.137*	0.136*
	(2.014)	(0.042)	(0.031)	(0.023)
Pistoia	12.075*	0.242*	0.136*	0.125*
	(2.193)	(0.029)	(0.020)	(0.014)
Firenze	8.976	0.215	0.137	0.107
	(1.242)	(0.032)	(0.023)	(0.017)
Livorno	12.236*	0.243*	0.136*	0.126*
	(2.319)	(0.029)	(0.019)	(0.017)
Pisa	12.589*	0.239*	0.136*	0.123*
	(1.994)	(0.036)	(0.027)	(0.020)
Arezzo	9.776*	0.219*	0.136*	0.108*
	(1.919)	(0.037)	(0.026)	(0.020)
Siena	11.106*	0.236*	0.136*	0.121*
	(1.826)	(0.038)	(0.028)	(0.020)
Grosseto	13.388*	0.259*	0.137*	0.137*
	(2.365)	(0.042)	(0.029)	(0.024)

The * denotes model-based estimate. Root mean squared error in parenthesis

**Fig. 10:**
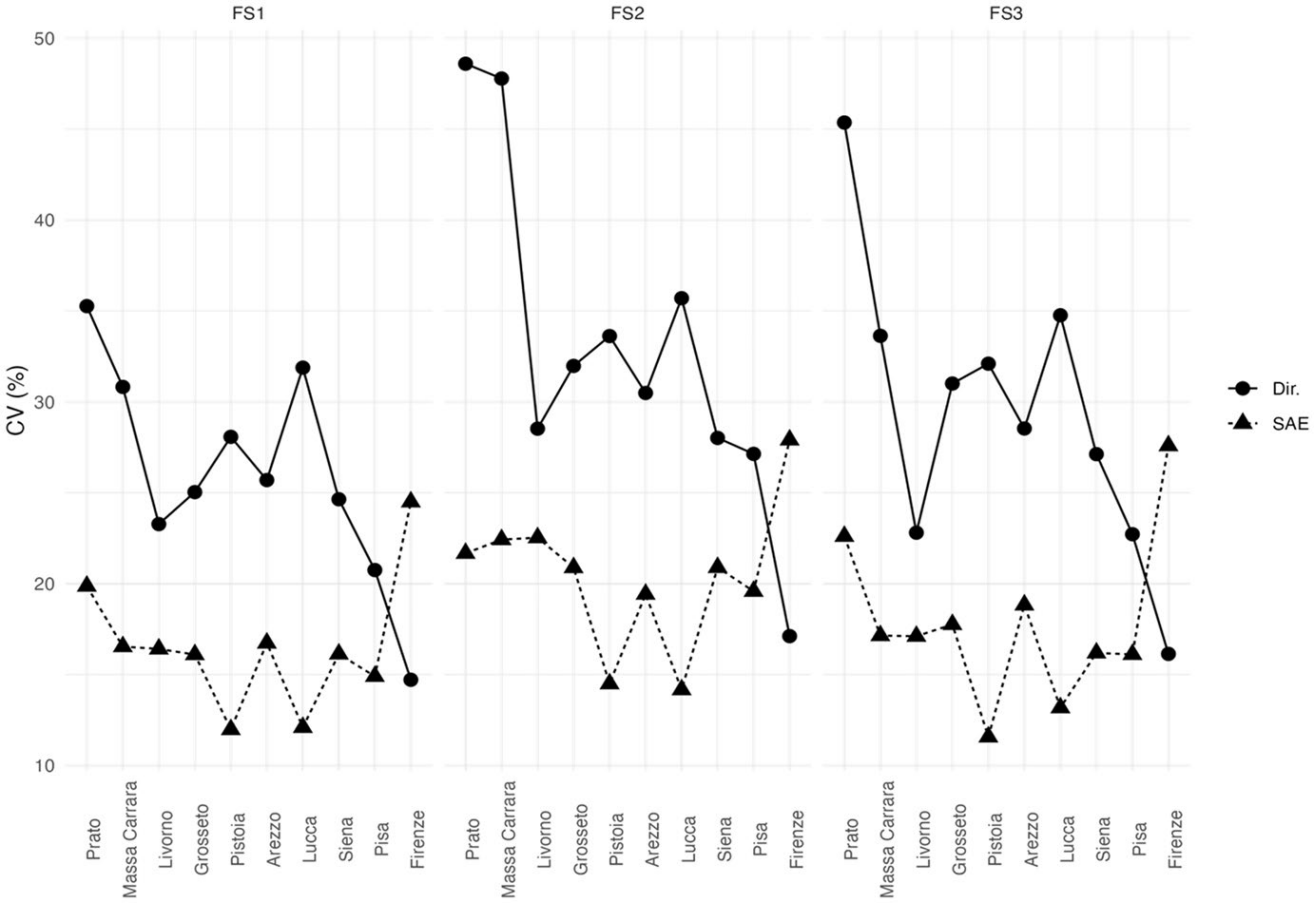
CVs of EBLUPs and direct estimators for each area (2021). Areas are sorted by increasing sample size

Regarding the FS measures, Fig. 10 shows the CVs of the direct estimates and the small area estimates. All the non-monetary poverty measures are estimated with a lower error in all the provinces considered with exception of Florence. The reason could be the variability of the indicators in Florence, indeed the variance of estimation is a function of the estimation itself, so that we expect larger uncertainty when the estimation focuses on rare events (Wolter 2007). However. the CVs of EBLUPs do not exceed 23% for any of the provinces (very good or good quality).

The auxiliary variables that we used to obtain small area estimates are the weighted 10-th percentile of the income distribution of the total income distribution (for HCR), and the percentage of households owning the house where they live (for FS1, FS2, FS3). Table 9 shows the final estimates at NUTS-3 level. As we did before, we report the most efficient estimate between the direct and the model-based estimate.

## Conclusions

In this paper, we estimated monetary and non-monetary poverty measures at two different levels of disaggregation in Tuscany. The data comes from a sample survey held by the Regional Institute for Economic Planning of Tuscany (IRPET), focusing on the economic and social features of the Tuscan households, with particular attention to the current economic situation (September 2021) and prospects.

In particular, we estimated the percentage of households living below the poverty line and three (fuzzy) supplementary measures of poverty that we identified in: deprivation in basic needs and social inclusion, children specific deprivation, and financial insecurity. Our results reveal the areas and the provinces with the major amount of people living in (monetary) poverty conditions, but also, they show that the perception of financial insecurity is dominating at each level of territorial disaggregation. Also, it reveals that children vulnerability is a problem that is similarly spread in Tuscany, while poverty in basic needs seems to have hit some areas/provinces more than others. Thus, measures related to monetary issues, as HCR and FS1 are those presenting more heterogeneity across provinces and across the six areas.

The importance of these findings is crucial from the point of view of local policy making, indeed specific addressed policy could reduce regional vulnerability and/or foster the capacity for resilience (Sánchez and Jiménez-Fernández 2022).

Having accurate measures at small area level is a crucial comprehensive information base for stakeholders and policy makers that can adopt the results as a starting point for the design of policies for the poor and vulnerable groups with the aim of i) addressing the gaps in social protection of Tuscan citizens and ii) making the regional welfare system more resilient to future shocks through more effective and flexible policies.

For these reasons we estimated the variance of the measures discussed as an assessment of their reliability. We noticed that in some areas/provinces, the estimated coefficients of variation were not so contained to guarantee a proper level of accuracy of the direct estimates.

For this reason, we used small area estimation to obtain new estimates with lower mean squared error. With this technique we were able to take almost all the estimates at a level more than acceptable. To state if an estimate is acceptable, we used the guidelines by Statistics Canada. However, some studies apply small area estimation regardless of the magnitudes of CVs (see Graf et al. 2019 as example).

Nevertheless, for some areas the small area gains were not always sufficient. These results may be in part because the number of areas considered is low so that the variance resulting from small area estimation may be overestimated. In principle, this is not a major problem if the variance is estimated in a conservative fashion. However, when the areas/province show a good level of accuracy we use the direct estimates as an official estimate. In fact, the properties of direct estimators are well-known while small area models may suffer from bias when the number of areas is small. We also noticed that in provinces with large sample size, the small area error tends to be greater. Situation like this have been already addressed in the literature and seem to be an effect of the large sample size.

## Footnotes

1 (http://www.toscanapromozione.it/uploads/documenti/Tuscany%20Regional%20Survey%20Gent.pdf; http://www.irpet.it/the-economy).

2 Computer Assisted Telephone Interview.

3 Computer Assisted Mobile Interview.

4 FCS method of the MI procedure of the SAS software.

5 Le povertà e l’inclusione sociale in Toscana—Quinto rapporto—anno 2021.

6 This value slightly differs from the estimate by Eurostat (12.4%).

7 This is Step 1 of the procedure outlined in Sect. 3.

8 The ratio of an estimate and its standard error. Usually expressed as a percentage. However, some studies have shown limitations of this measure (Kvålseth 2017).

9 https://www23.statcan.gc.ca/imdb/p2SV.pl?Function=getSurvey&SDDS=5232#a3.

## References

[CR1] Arima S, Datta GS, Liseo B (2015). Bayesian estimators for small area models when auxiliary information is measured with error. Scand. J. Stat..

[CR2] Bell WR, Chung HC, Datta GS, Franco C (2019). Measurement error in small area estimation: functional versus structural versus naïve models. Surv. Methodol..

[CR3] Benedetti I, Betti G, Crescenzi F (2020). Measuring child poverty and its uncertainty: a case study of 33 european countries. Sustainability (switzerland).

[CR4] Betti G, Verma V (2008). Fuzzy measures of the incidence of relative poverty and deprivation: a multi-dimensional perspective. Stat. Methods Appl..

[CR5] Betti G, Gagliardi F, Lemmi A, Verma V (2015). Comparative measures of multidimensional deprivation in the European Union. Empir. Econ..

[CR6] Betti, G., D’Agostino, A., Gagliardi, F., & Giusti, C.: The integrated fuzzy and relative index for poverty analysis: a review of applications in the social sciences. In Estudios de Economia Aplicada (Vol. 38. Issue 1) (2020). 10.25115/eae.v38i1.2985

[CR7] Carraro, A., Ferrone, L.: Measurement of multidimensional child poverty. In: Leal Filho. W., Azul. A., Brandli. L., Lange Salvia. A., Özuyar. P., Wall. T. (eds) No Poverty. Encyclopedia of the UN Sustainable Development Goals (2020). Springer.: Cham. 10.1007/978-3-319-69625-6_106-1

[CR8] Cerioli, A., Zani, S.: A Fuzzy Approach to the Measurement of Poverty (1990). 10.1007/978-3-642-84250-4_18

[CR9] Cheli B, Lemmi A (1995). A “totally” fuzzy and relative approach to the multidimensional analysis of poverty. Econ. Notes.

[CR100] EU (2021). https://ec.europa.eu/eurostat/web/nuts/background

[CR10] Fay RE, Herriot RA (1979). Estimates of income for small places: an application of james-stein procedures to census data. J. Am. Stat. Assoc..

[CR11] Graf M, Marín JM, Molina I (2019). A generalized mixed model for skewed distributions applied to small area estimation. TEST.

[CR12] Kvålseth TO (2017). Coefficient of variation: the second-order alternative. J. Appl. Stat..

[CR13] Molina I, Marhuenda Y (2015). Sae: an R package for small area estimation. R J..

[CR14] Prieto J (2022). A multidimensional approach to measuring economic insecurity: the case of Chile. Soc. Indic. Res..

[CR15] Rao, J.N.K., Molina, I.: Small Area Estimation: Second Edition (2015). 10.1002/9781118735855

[CR16] Ravallion, M.: Income inequality in the developing world. In *Science* (Vol. 344. Issue 6186) (2014). 10.1126/science.1251875 10.1126/science.1251875 24855260

[CR17] Ravallion, M.: Poor or just feeling poor? On using subjective data in measuring poverty. In Happiness and Economic Growth (2015). 10.1093/acprof:oso/9780198723653.003.0004

[CR18] Sánchez, A., Jiménez-Fernández, E.: European Union Cohesion Policy: socio-economic vulnerability of the regions and the COVID-19 shock. Appl. Res. Qual. Life (2022) 10.1007/s11482-022-10116-1 PMC9676792 36440458

[CR19] Tavares FF, Betti G (2021). The pandemic of poverty, vulnerability and COVID-19: evidence from a fuzzy multidimensional analysis of deprivations in Brazil. World Dev..

[CR20] Wolter, K.M.: Generalized variance functions. In: Introduction to Variance Estimation. Statistics for Social and Behavioral Sciences. Springer. New York. NY (2007). 10.1007/978-0-387-35099-8_7

